# A Case of Painless Legs and Moving Toes Syndrome in Parkinson's Disease Responsive to Dopaminergic Therapy

**DOI:** 10.1155/2016/6829410

**Published:** 2016-08-28

**Authors:** Sumihiro Kawajiri, Yasunobu Hoshino, Ryota Nakamura, Kazuyuki Noda, Yuji Tomizawa, Nobutaka Hattori, Yasuyuki Okuma

**Affiliations:** ^1^Department of Neurology, Juntendo University Shizuoka Hospital, Shizuoka, Japan; ^2^Institute of Oriental Medicine, Tokyo Women's Medical University, Tokyo, Japan; ^3^Department of Neurology, Juntendo University School of Medicine, Tokyo, Japan

## Abstract

Painless Legs and Moving Toes Syndrome (PoLMT) is a rare movement disorder characterized by flexion, extension, abduction, adduction, and torsion of toes without pain. It is considered a variant of Painful Legs and Moving Toes Syndrome (PLMT), which is characterized by similar movements but with pain. Although neuropathy and several central nervous system (CNS) involvements have been reported to be associated with PoLMT, the actual cause and mechanism remain unclear. Here we describe the first case of PoLMT in Parkinson's Disease (PD), parallel to parkinsonism in severity, who demonstrated a good response to dopaminergic therapy.

## 1. Introduction

Painful Legs and Moving Toes Syndrome (PLMT) is a rare continuous or semicontinuous involuntary movement disorder characterized by flexion, extension, abduction, adduction, and torsion of toes with pain in the affected extremity. On the other hand, Painless Legs and Moving Toes Syndrome (PoLMT) characterized by similar movements but without pain was discovered in 1993 and considered a variant of PLMT [1]. Although neuropathies, lumbosacral radiculopathy, and several central nervous system (CNS) involvements have been related with PLMT and PoLMT, the actual cause and pathophysiology is still unclear [2]. In this report, we present a case of PoLMT in Parkinson's Disease (PD), similar to parkinsonism in severity, with good response to dopaminergic therapy. We also present a corresponding video.

## 2. Case Presentation

We report a 72-year-old woman with past medical history of hypertension, spondylolisthesis, and cervical spondylosis. She developed resting tremor of the left lower limb 4 years before the onset of PoLMT. One year after onset of the tremor, she developed left dominant rigidity, akinesia, and resting tremor in the upper and lower limb. She was responsive to levodopa and thus diagnosed with PD. She had been stable with dopaminergic therapy within stage 3 of the Hoehn and Yahr (H-Y) scale. However, her parkinsonism progressed. In addition to continuation of treatment with levodopa (600 mg/day), entacapone (300 mg/day), and selegiline (5.0 mg/day), ropinirole (2 mg/day) was prescribed. Subsequently, postural abnormalities, such as dropped head and stooped posture, and other symptoms worsened to stage 4 of the H-Y scale. Subsequently, she was admitted to our hospital. The score on the Unified Parkinson's Disease Rating Scale (UPDRS) Part III was 45. She had mild hallucinations and anxiety but no dementia (28/30 on the Mini-Mental State Examination). Furthermore, she presented with repetitive involuntary moving toes on both sides in the absence of pain (first half of video). Brain MRI showed only mild ischemic changes. Whole spinal cord MRI revealed cervical spondylosis at C4–6 and spondylolisthesis at L4-5, as mentioned previously. Blood examination was almost within normal limits. Considering the form of the movement without pain and other clinical features, we diagnosed the movement as PoLMT. We discontinued ropinirole because we were concerned about aggravation of postural abnormality, which improved slightly. PoLMT appeared to be the same, and the H-Y score was still at 4. We began treatment with rotigotine (2 mg/day) and increased the dose to 10 mg/day. Then, parkinsonism improved to 30 of the UPDRS Part III score and stage 3 of the H-Y score. Surprisingly, PoLMT also improved markedly (latter half of video). She had been stable for several weeks; however, she developed severe hallucinations, and therefore rotigotine, selegiline, and entacapone were discontinued. Although her hallucinations resolved, both PoLMT and parkinsonism concurrently worsened. After 1 year, when she developed wearing-off complications, PoLMT also became worse at the off stage. Subsequently, we added entacapone and both parkinsonism and PoLMT improved at the off stage.

## 3. Discussion

PLMT is an uncommon illness described by Spillane et al. in 1971 [3]. According to a 76-case series report about PLMT [4], various movements were described, but they tended to be constant and to wax and wane in severity. Although peripheral neuropathy accounted for the most common cause of pain (28%), the cause in 42% of patients was not identified. PoLMT is assumed to be a variant of PLMT and is more uncommon, first reported by Walters et al. in 1993 [1]. To the best of our knowledge, only 12 cases have been reported so far. In addition to peripheral nerve involvement, CNS involvement (e.g., Wilson's disease, cerebral infarction, brain tumor, and spinal cord compression) has been reported [2]. With regard to treatment, if the etiology could be identified, treatment for primary diseases was preferred. If primary diseases could not be identified, quetiapine, gabapentin, or low doses of clonazepam were reported to be effective [2]. Although the detailed mechanism remains unclear, some mechanisms are speculated; for example, alterations in afferent sensory information due to peripheral nerve damage can cause subsequent reorganization of efferent motor activity [2]. In contrast, there are some reports suggesting that a dystonic mechanism contributes to this movement [2]. Our case developed not only PoLMT but also dropped head and stooped posture, which seemed to suggest dystonia. PoLMT in our case appeared to be tremulous and was similar to parkinsonism in severity. Dopamine agonists are reported to be effective for movements of some PLMT patients [4]. From these points of view, we could also consider PoLMT as a rare manifestation of parkinsonism and of wearing-off phenomena.

On the other hand, rotigotine in our case markedly improved PoLMT, while ropinirole exacerbated it. Pharmacologically, rotigotine acts as a dopamine receptor agonist with a higher affinity for the dopamine D1 receptor than ropinirole's [5]. Dopamine D1 receptors regulate neuronal growth and development and modulate dopamine receptor D2-mediated events. Dopamine D1 receptors may contribute to improvement of PoLMT through these functions.

It is reported that spinal compression can cause PoLMT [2]. Our case had cervical spondylosis and spondylolisthesis. However, PoLMT similar to parkinsonism in severity indicates that these are not direct causes, although they could be risk factors.

To the best of our knowledge, this is the first case of PoLMT in PD, parallel to parkinsonism in severity, that showed significant improvement by dopaminergic therapy. Although the mechanism remains unclear, our case suggests that PoLMT is a rare manifestation of parkinsonism and of wearing-off phenomena in particular. Further investigations are needed to reach verifiable conclusions.

## Supplementary Material

First half: Three days after ropinirole was discontinued and 1 day before rotigotine was initiated, the patient's bilateral toes and feet were involuntarily, continuously, and non-rhythmically, moving up and down at rest. Second half: Several days after the dose of rotigotine was increased to 10 mg/day, the movements decreased markedly, and continued only for a few seconds at rest.
